# Comparative genomic analysis of the gut bacterium *Bifidobacterium longum *reveals loci susceptible to deletion during pure culture growth

**DOI:** 10.1186/1471-2164-9-247

**Published:** 2008-05-27

**Authors:** Ju-Hoon Lee, VN Karamychev, SA Kozyavkin, D Mills, AR Pavlov, NV Pavlova, NN Polouchine, PM Richardson, VV Shakhova, AI Slesarev, B Weimer, DJ O'Sullivan

**Affiliations:** 1 Department of Food Science and Nutrition, Center for Microbial and Plant Genomics, University of Minnesota, 1500 Gortner Ave., St. Paul, MN 55108, USA; 2 Fidelity Systems Inc., 7961 Cessna Ave., Gaithersburg, MD 20879, USA; 3 Department of Viticulture and Enology, University of California, One Shields Ave., Davis, CA 95616, USA; 4 U.S. Department of Energy Joint Genome Institute, 2800 Mitchell Drive, Walnut Creek, CA 94598, USA; 5 Departments of Biology, Nutrition and Food Science, Center for Integrated BioSystems, Utah State University, 4700 Old Main Hill, Logan, UT 84322, USA

## Abstract

**Background:**

Bifidobacteria are frequently proposed to be associated with good intestinal health primarily because of their overriding dominance in the feces of breast fed infants. However, clinical feeding studies with exogenous bifidobacteria show they don't remain in the intestine, suggesting they may lose competitive fitness when grown outside the gut.

**Results:**

To further the understanding of genetic attenuation that may be occurring in bifidobacteria cultures, we obtained the complete genome sequence of an intestinal isolate, *Bifidobacterium longum *DJO10A that was minimally cultured in the laboratory, and compared it to that of a culture collection strain, *B. longum *NCC2705. This comparison revealed colinear genomes that exhibited high sequence identity, except for the presence of 17 unique DNA regions in strain DJO10A and six in strain NCC2705. While the majority of these unique regions encoded proteins of diverse function, eight from the DJO10A genome and one from NCC2705, encoded gene clusters predicted to be involved in diverse traits pertinent to the human intestinal environment, specifically oligosaccharide and polyol utilization, arsenic resistance and lantibiotic production. Seven of these unique regions were suggested by a base deviation index analysis to have been precisely deleted from strain NCC2705 and this is substantiated by a DNA remnant from within one of the regions still remaining in the genome of NCC2705 at the same locus. This targeted loss of genomic regions was experimentally validated when growth of the intestinal *B. longum *in the laboratory for 1,000 generations resulted in two large deletions, one in a lantibiotic encoding region, analogous to a predicted deletion event for NCC2705. A simulated fecal growth study showed a significant reduced competitive ability of this deletion strain against *Clostridium difficile *and *E. coli*. The deleted region was between two IS*30 *elements which were experimentally demonstrated to be hyperactive within the genome. The other deleted region bordered a novel class of mobile elements, termed mobile integrase cassettes (MIC) substantiating the likely role of these elements in genome deletion events.

**Conclusion:**

Deletion of genomic regions, often facilitated by mobile elements, allows bifidobacteria to adapt to fermentation environments in a very rapid manner (2 genome deletions per 1,000 generations) and the concomitant loss of possible competitive abilities in the gut.

## Background

Recent molecular studies into the microbial diversity of the human intestine reveal a much greater diversity than previously recognized and very little is currently known of the contribution of individual groups to the human organism [1]. One numerically dominant group of microbes, the bifidobacteria, is often suggested to be associated with good intestinal health given their overriding dominance in the feces of breast fed infants [2]. This phenomenon led to their discovery in 1899 by the pediatrician Henri Tissier and his subsequent use of these bacteria for the treatment of infantile diarrhea [3]. The proposed beneficial effect of bifidobacteria is further supported by the decrease of these bacteria in geriatric individuals and the concomitant increase of other microbial groups, most notably clostridia and *E. coli *[4-6]. This has led to the growing worldwide interest of including bifidobacteria in foods specifically for their potential intestinal health benefits [7]. However, clinical feeding studies with bifidobacteria show that while the strains can be detected in subject's feces during feeding trials, they are rapidly lost upon cessation of the studies pointing to a possible loss of competitive fitness of the strains for competition within the human intestinal environment [7-9]. This may be due to attenuation of the strains, as the fermentation environment is very different to the buffered and anaerobic environment of the human colon.

To further the understanding of genetic attenuation that may occur in bifidobacteria, the complete genomic sequence of a numerically dominant human intestinal isolate of *Bifidobacterium longum*, that was grown for less than 20 generations in laboratory media, was deciphered. This was compared to an available genome sequence of a culture collection strain, *B. longum *NCC2705 [10] and analysis of the functional attributes of its unique sequences have contributed to a better understanding of attenuation that can occur in these bacteria in a fermentation environment.

## Results and Discussion

### Genomic sequencing of a minimally cultured *B. longum *strain

**Figure 1:**
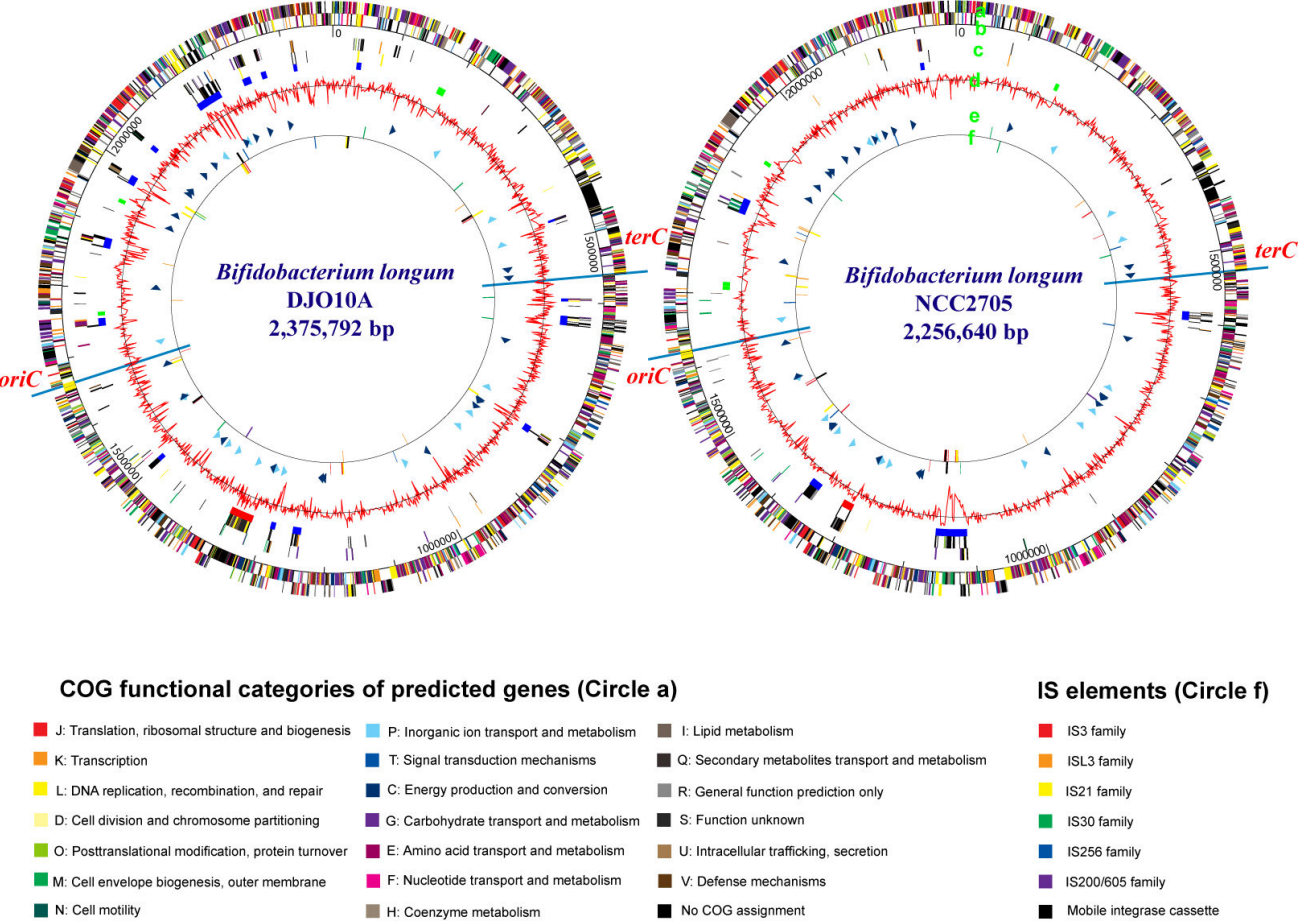
(**A**) *B. longum *strain DJO10A (**B**) NCC2705. Circle (a) indicates the coding regions by strand. The color of each gene refers to the COG functional categories. Circle (b) lists the number of base pairs (bp). Circle (c) contains the unique genes organized according to the coding strand (first two blocks) with the third block indicating the larger unique regions as defined in the text (blue), a prophage region (red), and rRNA operons (green). Circle (d) illustrates the G+C content. Circle (e) shows tRNA genes with block arrows indicating their coding strand. Circle (f) indicates insertion sequences (IS).

The power of comparative genomics can provide insights into features that are important for a species to survive and compete in its habitat. The genome sequence of the culture collection strain, *B. longum *NCC2705 [10], is available and the ability to compare this genome with one from a strain that was deliberately minimally cultured in vitro may provide new insights to features that may be important for this prominent species from the human large gut. Newly isolated and minimally cultured *B. longum *strains were characterized and strain DJO10A was selected based on its prominent ability to bacteriostatically inhibit other bacteria through the production of siderophores [11], a characteristic that appeared attenuated in all culture collection and commercial bifidobacteria analyzed. It was therefore selected for genomic sequencing as an isolate that likely had minimal attenuation from its origin in the intestine. The complete genome sequence of this strain was deciphered and consisted of one circular chromosome (Fig. 1) and two cryptic plasmids, pDOJH10L and pDOJH10S that were described previously [12].

### General characteristics of the *B. longum *DJO10A genome

The chromosome of *B. longum *DJO10A contained 2,375,792 bp, with 60.15% G+C content and 1,990 encoded genes containing four rRNA operons, 58 tRNAs, 6 insertion sequence (IS) families as well as one prophage (Table 1). Its genomic characteristics were analogous to strain NCC2705, except it contained an extra tRNA_Ser: GCT encoded on its prophage [13]. Codon usage analysis showed that this tRNA is the most frequently used tRNA_Ser in the prophage, while it is not the most used tRNA_Ser for the *B. longum *DJO10A genome (Additional file 1), pointing to an evolutionary selective pressure for its presence on the prophage. While both genomes contained tRNA's for every amino acid, the corresponding genes for aminoacyl-tRNA synthetases for both asparagine and glutamine are missing, suggesting a reliance on alternative pathways for translation with these amino acids, similar to many other bacteria [14,16]. Both these alternative pathways involve *gatABC*, which is present in both genomes as well as *gltX *and *aspS *involved in the glutamine and asparagine alternative translation pathways respectively, substantiating this proposed translation route. Interestingly, the *B. longum *genome contains novel mobile integrase cassettes (MIC) consisting of three different contiguous integrases flanked by an inverted repeat and a palindrome structure sandwiched by two IS*3*-type IS elements (Additional file 2). Analysis of the genome of *B. longum *NCC2705 revealed three analogous MIC elements, located in a non-linear fashion relative to strain DJO10A indicating these elements are indeed mobile (Additional file 3B). Interestingly, analysis of the genome sequences of another *Bifidobacterium *species, *B. adolescentis *(GeneBank AP009256), as well as other intestinal bacteria, *Bacteroides *(AE015928), *Lactobacillus *(CP000033), and *E. coli *(U00096), did not reveal MIC elements, suggesting these structures may be unique to a subset of closely related bifidobacteria.

**Table 1:** Overall characteristics of the genomes of *B. longum *strains DJO10A and NCC2705.

	**DJO10A**	**NCC2705**
	
Size of chromosome (bp)	2,375,792	2,256,640
Overall G+C %	60.15	60.12
Number of plasmids	2 (10 and 3.6 kb)	1 (3.6 kb)
		
**Genes**		
Total genes	1990	1727
Average gene length (bp)	1031	1115
Gene density (genes/kb)	0.838	0.765
Gene coding percentage (%)	86.4	85.3
Gene G+C %	61.13	60.86
		
**Unique Sequences**		
Strain-specific unique genes	269	117
Number of unique regions^a^	17	6
Number of genes in unique regions	218	84
Prophage	1	1
Number of genes in prophage region	57	19
		
**RNAs and Repeat Sequences**		
rRNA operons	4	4
tRNAs	58	57
Tandem repeats	22	23
		
**Mobile Elements^b^**		
Mobile integrase cassette (MIC)	4	3
IS3 family	13	14
IS21 family	10	7
IS30 family	9	5
IS256 family	4	7
ISL3 family	7	12
IS200/605 family	1	1

^a^, refers to unique regions that encode functional or hypothetical genes in DNA regions > 3 kb, ^b^, includes fragmented elements

### Organization of the origin and terminus of replication

An *oriC *and *terC *were found in identical locations in the genome of strain DJO10A and the updated genome sequence of strain NCC2705 (Additional file 4). These regions are extremely highly conserved in both genomes (> 99.9% identity) and consist of three *oriC *clusters and a *terC*, which is consistent with the predicted replication regions from other bacterial genomes [16]. However, the location of the observed *oriC *region in both genomes is slightly different from the predicted location based on genome asymmetry, a feature that has previously been seen in the *Helicobacter pylori *26695 genome [16,17]. As well as the multiple *oriC *clusters, there are 7 different types of DnaA boxes, consistent with the majority of sequenced genomes and are proposed to be involved in controlling initiation of chromosome replication [16].

### Restriction and modification (R-M) systems

The protective role that R-M systems impart on bacteria has been compared to the immune system of higher organisms [18]. The presence of these systems in numerous bacteria demonstrates their important role for bacterial survival in nature. Both of the *B. longum *genomes encode type I and two type II R-M systems that are highly conserved (Additional file 5). They also contain an Mrr system that is predicted to restrict methylated DNA (usually *Hha*II or *Pst*I methylated DNA) that is 100% conserved between both strains (Additional file 5A). The clustering of Mrr with the type I R-M system is similar to *E. coli *K12 (GenBank U00096). The low identity (40%) between the HsdS proteins in the two strains likely reflects the independent evolution of this type I R-M system in these strains following their evolutionary divergence, as these systems evolve by changing their specificity components (HsdS) to enable it to recognize different sequences. This is substantiated by the existence of an *hsdS *gene that was inactivated by an IS*256 *insertion event and both parts of this disrupted gene exhibit much higher conservation, suggesting the insertion event occurred before their evolutionary divergence (Additional file 5A). Upstream from this locus in strain DJO10A there is another restriction gene, McrA (restricts DNA methylated by *Hpa*II or *Sss*I), that is not present in NCC2705. The conserved type II R-M systems in both strains are isoschizomers of *Sau*3AI and *Eco*RII which restrict DNA at very frequently occurring sites (Additional file 5B and 5C). This, together with the range of restriction systems present, may be a factor in limiting the incursion of foreign DNA into these bacteria thus explaining the very low electroporation frequencies reported for bifidobacteria to date.

### Unique genome regions in the *B. longum *strains

**Figure 2:**
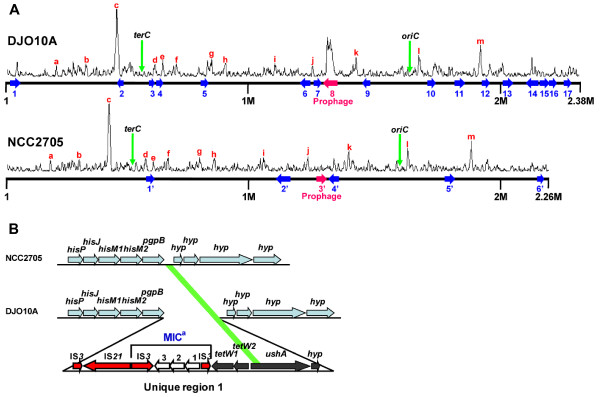
Genome unique regions. **(A)** Base deviation index (BDI) analysis of the *B. longum *DJO10A and NCC2705 genomes. Unique regions of each genome as defined in the text are numbered. The locations of *oriC *and *terC *are indicated by green arrows. Letters refer to predicted gene phenotypes from regions with definitive BDI peaks that are present in both genomes, a, GTPase, b, cation transport ATPase, c, DNA partitioning protein, d, choloylglycine hydrolase, e, glutamine synthase beta chain, f, alanyl-tRNA synthetase, g, pyruvate kinase, h, cation transport ATPase, I, fibronectin type III, j, aminopeptidase C, k, subtilisin-like serine protease, l, sortase, m, fatty acid synthase. **(B)** Organization of the unique region 1 showing the location of a 361 bp DNA remnant, indicated by the green bar, from the *ushA *gene remaining at the predicted deletion location in NCC2705. Sky blue colored ORFs indicate common genes between both genomes. ^a^, mobile integrase cassette.

Alignment of the genome sequence of *B. longum *DJO10A with that of strain NCC2705 illustrates that they are highly conserved and collinear, except for the mobile IS and MIC elements (Additional file 3). There is also an apparent genome reduction in strain NCC2705, consistent with previous observations for microbes growing in a stable environment without horizontal gene transfer opportunities and redundant genes accumulating mutations before subsequent deletion [19]. There are 248 unique sequences of > 10 bp between the two genomes, with the majority of them being short and encoding few if any genes. This high number of unique sequences between the two strains was surprising given that the genomes of a clinical isolate of *Mycobacterium tuberculosis *and one that was extensively passaged for decades in the laboratory display only 86 of such regions in genomes twice the size [20]. There are 23 larger unique regions that encode functional or hypothetical genes and range in size from 3.0 to 48.6 kb, with 17 of these unique regions present in strain DJO10A encoding 219 predicted genes, and 6 unique regions in NCC2705 encoding 84 genes (Fig. 2A). These unique regions are not clustered around the *oriC *and *terC *which have previously been associated with regions of intraspecies variation [21,22].

One unique region in each genome corresponds to a prophage. The prophage in strain NCC2705, which is truncated, appears to be a longtime resident of the genome as it does not correspond with a Base Deviation Index (BDI) peak (Fig. 2A), as this analysis predicts recent horizontal gene transfer (HGT) events. This appears to have been replaced in the genome of strain DJO10A with a different prophage, that is complete and inducible [12] and corresponds with a significant BDI peak substantiating this recent HGT event. The other five unique regions in strain NCC2705 contain largely hypothetical genes or genes of diverse functions without any significant gene clusters. However one of these regions (unique region 4') does encode putative xylan degradation genes, which is a function predicted to be important for competition in the large intestine. As this region corresponds to a BDI peak, it suggests it may be a recent acquisition by this strain and its evolution in the large intestine would provide the selective pressure for acquiring this unique region. Of the other 16 unique regions in the strain DJO10A, eight encode significant gene clusters involved in functions predicted to be important for competition in the large intestine, specifically oligosaccharide and polyol utilization, arsenic resistance and lantibiotic production.

### Oligosaccharide and polyol utilization

**Figure 3:**
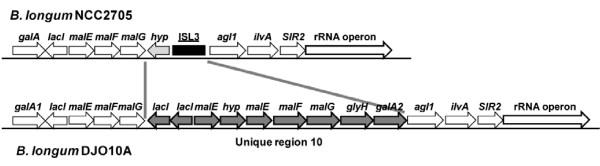
**Comparsion of oligosaccharide utilization gene cluster 7 between two *B. longum *genomes.** DJO10A-unique genes in unique region 10 are colored dark grey, ISL3-type IS element is colored black and other matched genes are colored white. *galA*, α-galactosidase; *lacI*, LacI-type repressor; *malEFG*, ABC-type transport system; ISL3, ISL3-type IS element; *agl1*, glycosidase; *ilvA*, threonine dehydratase; *SIR2*, NAD-dependent protein deacetylase; glyH, glycosyl hydrolase; *hyp*, hypothetical protein.

According to a COG functional classification [23], the highest number of unique genes in strain DJO10A with a predicted function belongs to the carbohydrate metabolism [G] category (Additional file 6). Interestingly, most of the unique genes in the carbohydrate metabolism category are involved in oligosaccharide utilization, which is the major carbohydrate source available to microbes in the large intestine. In all there are 11 oligosaccharide utilization gene clusters in strain DJO10A, of which 5 are fully present and 2 are partially present in strain NCC2705 (Additional file 7). It is noteworthy that one of these clusters (cluster 7 in Additional file 7) contains an ISL*3 *element in the NCC2705 genome at the precise location of the extra oligosaccharide utilization genes in strain DJO10A (Fig. 3). A BDI analysis suggested that the extra oligosaccharide gene clusters in strain DJO10A were not acquired following evolutionary divergence from strain NCC2705, as neither corresponds with a BDI peak (Fig. 2A). The majority of BDI peaks suggesting recent HGT events were the same in both genomes substantiating this analysis. This would suggest the six unique regions (6, 9, 10, 11, 15 and 17) encoding oligosaccharide utilization genes were likely lost from strain NCC2705 during its adaptation to a fermentation environment. Further evidence for the loss of these unique regions from strain NCC2705 comes from a DNA remnant of 361 bp (98% identity) from the *ushA *gene within the unique region 1 that was left remaining at this locus in NCC2705 (Fig. 2B).

**Figure 4:**
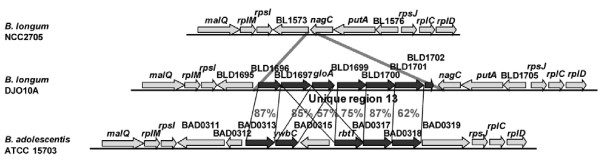
**Organization of genes involved in polyol metabolism in the unique region 13 in strain DJO10A and comparison with an analogous region in *B. adolescentis *ATCC 15703.** Amino acid identities are indicated between homologous genes. ORFs shaded black are from unique region 13 and corresponding homologs in *B. adolescentis *ATCC 15703.

Polyols are not digestible by humans and their metabolism is believed to be important for bacterial competition in the human large intestine and their ingestion has been implicated in increased bifidobacteria numbers [24]. While strain NCC2705 does not contain genes involved in polyol metabolism, unique region 13 of strain DJO10A is dedicated to this (Fig. 4), containing genes involved in polyol recognition, transport and dehydration, and there are also some polyol metabolism genes in unique region 11. Given that unique region 13 does coincide with a BDI peak (Fig. 2A), it may represent gene acquisition by strain DJO10A. Interestingly, a similar polyol locus was found in *B. adolescentis *ATCC 15703 at a similar genome location (Fig. 4).

### Arsenic resistance

**Figure 5:**
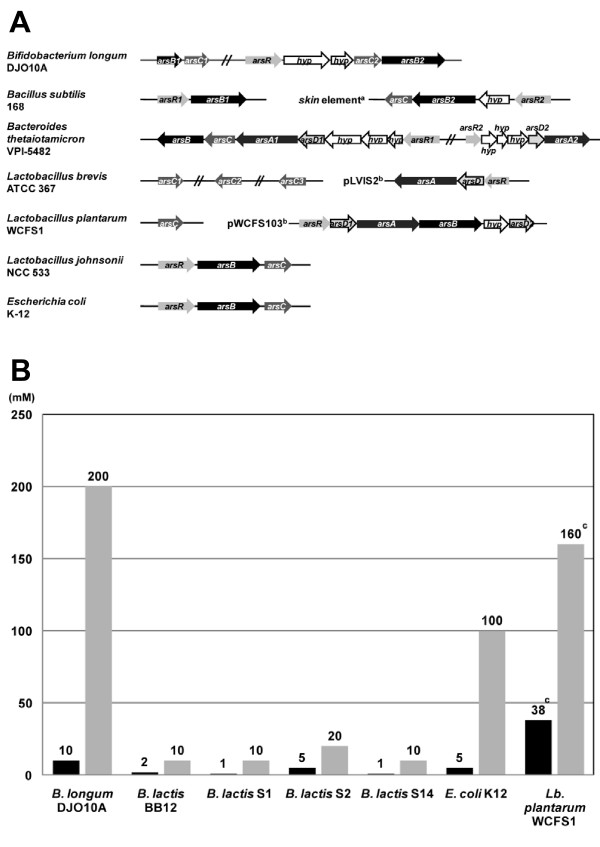
Arsenic resistance of selected bacteria. **(A)** Genetic organization of arsenic resistance gene clusters compiled from the completed genome sequences of *Bifidobacterium longun *DJO10A, *Bacillus subtilis *168 [34], *Bacteroides thetaiotamicron *VPI-5482 [35], *Lactobacillus brevis *ATCC 367 [36], *L. plantarum *WCFS1 [37], *L. johnsonii *NCC 533 [38] and *E. coli *K-12 [39]. ^a^, 48 kb element that is excised by the site-specific recombinase SpoIVCA during sporulation, ^b^, indicates a plasmid sequence, *arsR*, repressor, *arsA*, arsenite stimulated ATPase, *arsB*, arsenite efflux pump, *arsC*, arsenate reductase, *arsD*, arsenic chaperone, *hyp*, hypothetical protein. **(B)** Comparison of arsenic resistance activity in *B. longum *DJO10A with fermentation adapted *B. animalis *subsp. *lactis *strains, *E. coli *and *Lactobacillus plantarum*. ^c^, calculated from data presented in van Kranenburg et al., [40].

Other unique regions in strain DJO10A encode gene clusters predicted to be involved in characteristics that would be important for survival and competition in the human intestine. Two operons encoding ATP-dependent arsenic resistance genes are in unique regions 5 and 7 and may be important for intestinal survival as the human intestine contains low concentrations of arsenic from the diet [25]. Many intestinal bacteria such as *E. coli, Lactobacillus *and *Bacteroides *contain arsenic resistance genes (Fig. 5A), substantiating the competitive advantage for having this ability in the intestine. As the unique regions, 5 and 7, containing these arsenic resistance genes do not correspond to BDI peaks (Fig. 2A), it suggests they may not be recently acquired by strain DJO10A, but rather lost from strain NCC2705. This theory, that adaptation to a pure-culture fermentation environment can result in loss of arsenic resistance, was further substantiated by the exceptional arsenate resistance of strain DJO10A which was 2,000% greater than a fermentation adapted *Bifidobacterium *isolate (*B. animalis *subsp.*lactis *BB12) and 100% greater than *E. coli *K12 (Fig. 5B). This would suggest that this phenotype is a competitive advantage to intestinal isolates, but not of significance for pure-culture fermentation environments.

### Lantibiotic production

**Figure 6:**
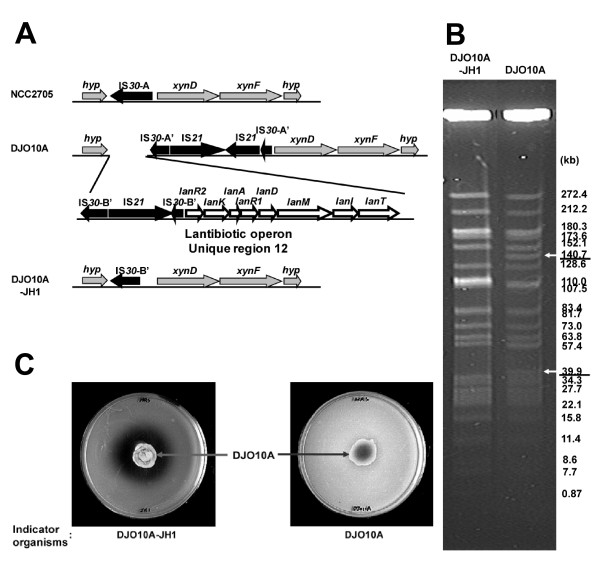
Lantibiotic prodiction by *B. longum *DJO10A. **(A)** Organization of the lantibiotic encoding unique region 12 of *B. longum *DJO10A and the corresponding genome locations in strains NCC2705 and DJO10A-JH1. The A or B designator following IS*30 *refer to unique classes of IS*30 *elements that are only found at this location in the genome. The ' designator indicates a fragmented IS*30 *element. **(B)** Pulsed Field Gel Electrophoresis (PFGE) analysis of *Xba*I-digested total DNA from *B. longum *DJO10A and its fermentation adapted isolate, DJO10A-JH1. White arrows indicate bands missing from strain DJO10A-JH1. **(C)** Bioassay for lantibiotic production by *B. longum *DJO10A with strains DJO10A and DJO10A-JH1 as indicator bacteria.

The production of antimicrobial peptides, or bacteriocins, is an important characteristic for bacterial competition in natural environments. One exceptionally broad spectrum class of bacteriocins is the lantibiotics, which are post-translationally modified to form lanthionine residues and to date none have been isolated from any bifidobacteria. A 10.2 kb gene cluster encoding all the genes indicative of a lantibiotic was detected in the unique region 12 of strain DJO10A (Fig. 6A). It was also noted that this unique region did not correspond to a BDI peak, suggesting a likely loss of this region from strain NCC2705. As lantibiotic production would be very advantageous for microbial competition in the intestine, but of no value to a microbe in pure culture, it provides the selective pressure for the loss of this unique region 12 from strain NCC2705.

### Genome attenuation of *B. longum *in fermentation environments

Given the large number of unique DNA regions in the genome of strain DJO10A, that are predicted to have been lost from strain NCC2705, it suggests that deletion of DNA regions that are not useful may reflect the rapid adaptation of *B. longum *to a new and very different environment than exists in the human large gut. This would suggest an elevated mutation frequency. A comparative nucleotide substitution analysis between strains DJO10A and NCC2705 shows the majority of genes are highly conserved (Additional file 8), which is to be expected with two closely related strains. However, analysis of the 52 least conserved genes (listed as 'positive selection' in Additional file 8) indicates that of the mutations that can be attributed to one strain or the other (frameshifts, deletions, insertions and stop mutations), 11 are from strain NCC2705 and three from strain DJO10A (Additional file 9). Further substantiation of an increased mutation frequency in strain NCC2705 comes from comparing genes encoding surface protein homologs between the two strains. A search of the DJO10A genome for LPXTG motifs, which is a signature of one class of cell surface anchoring proteins found four potential proteins and SignalP analysis of these proteins (BLD1468, BLD1511, BLD1637 and BLD1638) confirmed the presence of a signal sequence in each case (Additional file 10). In addition, BLASTP analysis of these four proteins showed that they are very similar to other known surface proteins containing the LPXTG motif. The NCC2705 showed three of these gene homologs (BLD1468, BLD1637 and BLD1638), and had a predicted protein exhibiting 99% amino acid identity to BLD1511, but was missing the LPXTG motif due to an ISL3 insertion in the 3' end of the gene. This further highlights the rapid evolutionary status of bifidobacteria when they are removed from the human gut into pure-culture fermentation environments.

### IS30 'jumping' in the *B. longum *genome

**Figure 7:**
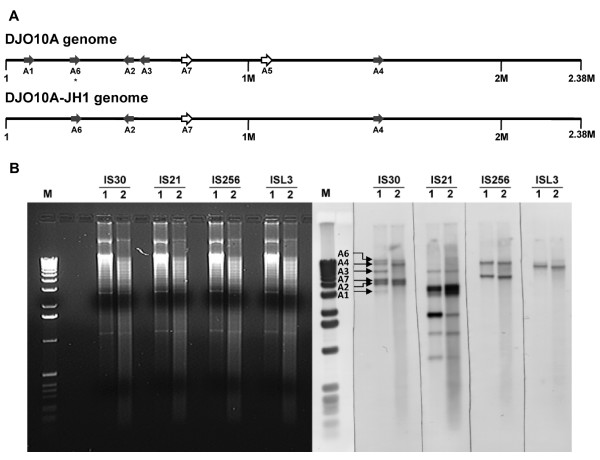
IS*30 *'jumping' in the genome of *B. longum *DJO10A. **(A)** Genome positioning of the IS*30 *elements in the genome of *B. longum *DJO10A and the laboratory adapted strain DJO10A-JH1. The gray arrows indicate the five elements identified by direct sequencing of DJO10A genomic DNA. The white arrows indicate the location of elements that were detected in some sequencing clones prepared from DJO10A genomic DNA. The asterisk under A6 indicates this element was missing from some sequencing clones of DJO10A DNA. **(B)***Nru*I digested genomic DNA from DJO10A shown in the left gel and its Southern hybridization (right gel) using probes specific for four different IS element families. (1) refers to DJO10A and (2) refers to DJO10A-JH1. Arrows indicate bands in DJO10A corresponding to specific IS*30 *elements as illustrated in (A).

The dynamic environment within the *B. longum *cell in a fermentation environment is further substantiated by the intriguing observation during genome sequencing from different batches of DNA that everything was identical except for the location of some IS*30 *elements (Fig. 7A). This very rapid movement of an IS element within a cell has not been observed previously. The movement of IS*30 *within the genome occurred only at specific sites, consistent with its insertion target specificity [26].

### Adaptation of *B. longum *DJO10A to a pure-culture environment

To test the hypothesis that the switch from a variable and complex environment like the gut to a relatively stable and simplified, pure-culture one, results in hyper IS*30 *activity and rapid DNA loss of regions that are no longer beneficial to the new environment, strain DJO10A was grown in a typical laboratory medium without pH control for ~1,000 generations. Isolated colonies were then screened for seven unique regions encoding functions predicted to be useful for survival in the human gut. One of these regions (no. 12) involved in the lantibiotic production was found to be missing from 40% of the isolates (Additional file 11) substantiating this hypothesis. Analysis of this adapted strain, DJO10A-JH1, shows the deletion extended over the full lantibiotic region very similar to strain NCC2705 (Fig. 6A). It is further noted using Pulsed Field Gel Electrophoresis (PFGE) that the 39.9 kb *Xba*I band containing this region is missing from strain DJO10A-JH1 (Fig. 6B). The loss of the complete lantibiotic gene cluster from 40% of the culture was intriguing as the cluster also encodes the immunity gene to protect cells from the lantibiotic activity. However, analysis of lantibiotic production by strain DJO10A showed that none occurred during growth in broth media, and a solid surface such as agar, was needed for production (Fig. 6C) similar to streptin production from *Streptococcus pyogenes *[27]. The loss of the complete lantibiotic gene cluster renders strain DJO10A-JH1 sensitive to this pronase-E sensitive lantibiotic, which is also active against a wide spectrum of bacteria (Fig. 6C). Interestingly, the lantibiotic genome region that was deleted during the adaptation of strain DJO10A to the pure-culture environment was located between two IS*30 *elements, suggesting its role in genome deletion events.

It was also noted that the pure-culture adapted strain, DJO10A-JH1, was also missing a 140.7 kb *Xba*I band (Fig. 6B). It is intriguing that this band contains one of the four MIC elements, suggesting it may have been involved. PCR analysis of the loci immediately bordering this MIC element revealed the deletion extended between 10 and 50 kb directly downstream from this element substantiating its likely role in this deletion event. This further substantiated the rapid loss of DNA, and the prominent role of mobile elements, during evolutionary adaptation by these bacteria.

Southern hybridization of strains DJO10A and DJO10A-JH1 substantiate the IS*30 *'jumping' during growth in a pure-culture environment, while the positions of the other IS elements (IS*21*, IS*256 *and ISL3) remained stable (Fig. 7B). This IS*30 *hyperactivity in *B. longum *genomes may play an important role in deletion events and genome reduction during adaptation to new environments.

### Competitive 'fitness' of the adapted *B. longum *strainDJO1A-JH1

The rapid genome reduction experienced by *B. longum *DJO10A during pure-culture growth in fermentation conditions suggested that the genomic regions lost may have been important for competition in the intestine. To test if this in vitro adaptation affected the 'fitness' of the strain, a simulated fecal competitive approach was developed. Bifidobacteria are frequently proposed to successfully compete against members of the clostridia and the enterobacteriae in the intestinal environment. A member of both of these bacterial groups was selected to test the relative competitive abilities of *B. longum *DJO10A and its in vitro adapted derivative, strain DJO10A-JH1. To ensure that the selected competitor strains were not attenuated in any way, new isolates were obtained from fresh feces by plating on selective media and speciated using a sequence analysis of the 16S rRNA gene. This resulted in the isolation of *Clostridium difficile *DJOcd1 and *E. coli *DJOec1, which were minimally cultured prior to undergoing fecal competitive experiments with the *B. longum *strains. An in vitro growth rate analysis established that *E. coli *DJOec1 had the fastest growth rate, followed by *C. difficile *DJOcd1, *B. longum *DJO10A-JH1 and *B. longum *DJO10A (Additional file 12). The noticeable increased growth rate of *B. longum *DJO10A-JH1 compared to strain DJO10A substantiated that the genome reduction of strain DJO10A-JH1 favored the in vitro growth environment.

**Figure 8:**
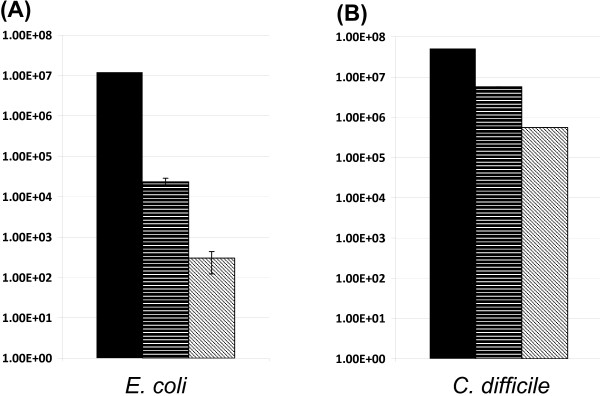
Simulated fecal competitive analysis of *B. longum *DJO10A and its in vitro adapted derivative, strain DJO10A-JH1, against *Clostridium difficile *and *E. coli*. **(A)** Viable cell counts of *E. coli *DJOec1 at the beginning of the competitive study (black), following competition with *B. longum *DJO10A-JH1 (horizontal lines) and *B. longum *DJO10A (hatched). **(B)** Viable cell counts of *C. difficile *DJOcd1 at the beginning of the competitive study (black), following competition with *B. longum *DJO10A-JH1 (horizontal lines) and *B. longum *DJO10A (hatched). N = 3.

Competitive growth experiments with both *E. coli *DJOec1 and *C. difficile *DJOcd1 in a simulated anaerobic fecal environment revealed that *B. longum *DJO10A had a significantly greater ability to compete against both *E. coli *and *C. difficile *(Fig 8). The significantly greater reduction in both these groups of bacteria by *B. longum *DJO10A supports the genome analysis hypothesis that the genome reduction exhibited in pure-culture growth may compromise a bacterium's ability to compete in its original environment.

While the simulated fecal competition studies suggested that the lantibiotic encoding genome region was important for competition in the human intestine, in vivo studies in an intestinal model would be necessary to verify this hypothesis.

## Conclusion

This study compares the genomic sequences of two strains of *B. longum *and suggests that bifidobacteria can rapidly loose genome regions during pure-culture growth that may be important for intestinal survival. This genomic prediction was experimentally validated during pure-culture growth of strain DJO10A and the genome reduction was shown to reduce competitive 'fitness' in a simulated fecal environment. The rapid loss of genomic regions that may be important for intestinal competition may compromise the ability of exogenous bifidobacteria to re-colonize human intestines.

## Methods

### Bacterial strains and growth conditions

*Bifidobacterium longum *strain DJO10A was isolated from a healthy young adult's feces [28] and *B. animalis *subsp. *lactis *BB12 was obtained from Chr. Hansen. *B. animalis *subsp. *lactis *strains S1, S2, and S14 are genetically distinct isolates from fermented foods (J.-H. Lee. and D.J.O'Sullivan, unpublished). *Clostridium difficile *DJOcd1 was isolated from fresh feces by plating on *Clostridium difficile *Selective Agar (BD Diagnostics) and speciated using a sequence analysis of its 16S rRNA gene. *E. coli *DJOec1 was obtained from fresh feces by plating on MacConkey agar (Difco) and speciated using a sequence analysis of its 16S rRNA gene. *E. coli *K12 was obtained from the American Type Culture Collection (ATCC). Bifidobacteria were cultivated at 37°C in MRS (Difco) supplemented with 0.05% L-cysteine·HCl (Sigma), Bifidobacteria Low-Iron Medium (BLIM) [28] or Bifidobacteria Fermentation Medium (BFM) (2% proteose peptone, 0.15% K_2_HPO_4_, 0.15% MgSO_4_·7H_2_O, 0.5% D-glucose) under anaerobic conditions using either the BBL Anaerobic system (BBL) or the Bactron II Anaerobic/Environmental Chamber (Sheldon Manufacturing).

### Genome sequencing and assembly

Whole-genome shotgun sequencing was carried out at the US Department of Energy Joint Genome Institute (JGI). Sequences were assembled into 227 contigs using the Phred/Phrep/Consed software and the sequence coverage was 9.2-fold. Gap closure and genome sequence finishing was carried out at Fidelity Systems using ThermoFidelase-Fimer direct genome sequencing technology [29]. Shotgun reads with and without IS*30 *elements covering A5, A6 and A7 loci were identified and assembled separately. The presence and location of long repeated sequences in genomic DNA samples were verified by direct genomic sequencing of the unique/repeat junctions. The resolution of the most complex high GC-rich repeats was achieved by sequencing of PCR products amplified with a hybrid TopoTaq DNA polymerase with increased strand displacement capacity.

### Bioinformatic analysis

Annotation of all open reading frames (ORFs) was carried out using Glimmer, GeneMark, JGI annotation tools and GAMOLA [30], before manual checking of all predicted genes. A comparative analysis of the two *B. longum *genomes was conducted using MUMmer3, ACT4 and ClustalX. The origin of replication and terminus were predicted using OriLoc [31]. Codon usage was analyzed using the General Codon Usage Analysis (GCUA) program [32]. The base-deviation index (BDI) was performed by scaled χ^2 ^analysis of Artemis8. To predict gene functions, the two conserved protein domain databases of GAMOLA and InterProScan were used. COG functional categories were used for functional classification of all genes in both *B. longum *genome sequences.

### Molecular techniques

General sequencing was conducted using a Big-Dye terminator and ABI Prism 3730*xl *Auto sequencer (Applied Biosystems). All PCR primers are listed in Additional file 13. For Southern blot analysis of unique region 12, a 646 bp probe from the *lanM *gene was obtained using PCR with LANT-F and LANT-R primers. Probes for IS elements were also PCR amplified. Probes were DIG-labeled and hybridized with digested genomic DNA according to the manufacturer's instructions (Roche). Pulsed field gel electrophoresis of *Xba*I-digested *B. longum *genomes was performed using a CHEF-DR III Variable Angle Pulsed Field Electrophoresis System according to manufacturer's instructions (Bio-Rad).

### Identification of gene homologs between the two *B. longum *genomes

Comparative nucleotide substitution analysis by Nei and Gojobori's algorithm [33] was used to identify gene homologs. The predicted genes of both genome sequences were compared using the local BlastN program in the NCBI toolkit and 1,590 aligned genes were used for the nucleotide substitution analysis by Nei's unweighted method I [33]. According to the ratio of dN:dS, all matched genes were categorized into three groups, highly conserved (< 0.035), normal, and positive selection (> 1).

### Minimal inhibitory concentration of arsenic

To determine the minimal inhibitory concentration of arsenic, BLIM was supplemented with different concentrations of sodium arsenite (AsO_2_^-^, 1 to 100 mM) and sodium arsenate (AsO_3_^-^, 1 to 500 mM). Freshly grown cultures were sub-inoculated into the arsenite/arsenate media and incubated anaerobically at 37°C for 48 h.

### Adaptation of *B. longum *DJO10A to in vitro fermentation conditions

*B. longum *DJO10A was grown in BFM continuously up to ~1,000 generations. The culture was then serially diluted and plated on BFM agar. Ten colonies were randomly selected for analysis.

### Mapping the deletions in strain DJO10A-JH1

To find the precise location of the deletion of the lantibiotic operon in the *B. longum *DJO10A-JH1 genome, PCR was used to test for several genes within the lantibiotic operon. The two primers F3 (position 1,974,570–1,974,587 bp) and R3 (position 1,996,024–1,996,005 bp) were used to amplify a ~1.8 kb region spanning the deletion and sequencing located the precise borders (Figure 6). To map the position of the deletion in the 140.7 kb *Xba*I fragment, primers MIC-F1 (position, 1,539,767–1,539,768) and MIC-R1 (position, 1,542,535–1,542,553) were used to amplify the upstream region of MIC III and primers MIC-F2 (position, 1,543,406–1,543,424) and MIC-R2 (position, 1,545,713–1,545,732) were used to amplify the downstream region.

### Bioassay for lantibiotic activity

*B. longum *DJO10A was inoculated into the center of an MRS agar plate and incubated anaerobically at 37°C for 2 days. After incubation, molten 0.5% top agar of the same medium containing 1% of an indicator strain was overlaid on the plates prior to incubation.

### Simulated fecal competitive analysis of bifidobacteria

To access the competitive 'fitness' of the wild-type *B. longum *DJO10A compared to its in vitro adapted derivative strain DJO10A-JH1, a simulated in vitro fecal system was developed. Triplicate experiments for each strain were used. Each experiment was conducted in 10 g sterilized feces in an anaerobic chamber, to which 0.38 g Reinforced Clostridial Medium (RCM) and 0.02 g mucin (Porcine gastric type III) was added. The two competitor bacteria were added to all tubes at calculated concentrations of 1.2 × 10^7 ^cfu/g for *E. coli *DJOec1 and 5.1 × 10^7 ^for *Clostridium difficile *DJOcd1. *B. longum *DJO10A was added to three tubes at a calculated concentration of 4.0 × 10^7 ^cfu/g and strain DJO10A-JH1 to the other three tubes at 4.4 cfu/g. Standard viable plate counts were used to calculate all bacterial concentrations. After thorough mixing in an anaerobic environment, the tubes were left at 37°C for 3 days, whereby the entire fecal samples were homogenized in 90 ml peptone water to conduct an accurate serial plate count analysis.

### Nucleotide sequence accession number

The Sequence and annotation data have been deposited in GenBank under the accession number CP000605.

## Authors' contributions

J-HL carried out the comparative and functional genomic analysis, and co-wrote the manuscript; VNK assembled the genome sequence of *B. longum *DJO10A into a single contig; SAK assembled the genome sequence of *B. longum *DJO10A into a single contig, observed the IS*30 *hyperactivity and critiqued the manuscript; DM facilitated the shotgun sequencing of *B. longum *DJO10A and critiqued the manuscript; NVP assembled the genome sequence of *B. longum *DJO10A into a single contig; NNP, assembled the genome sequence of *B. longum *DJO10A into a single contig; PMR carried out the shotgun sequencing of *B. longum *DJO10A; VVS assembled the genome sequence of *B. longum *DJO10A into a single contig; AIS assembled the genome sequence of *B. longum *DJO10A into a single contig; BW facilitated the shotgun sequencing of *B. longum *DJO10A; and DJO'S designed the study, analyzed the results and co-wrote the manuscript.

## Supplementary Material

Additional file 1 Comparison of serine codon usage between chromosomal and prophage genes in strain DJO10A. Click here for file

Additional file 2 Organization of mobile integrase cassettes (MIC) in *B. longum *DJO10A. (**A**) and NCC2705, (**B**). Orfs 1, 2 and 3 refer to three contiguous, but different *xerC *integrase genes. P, a conserved 20 bp palindrome (TTAAACCGACATCGGTTTAA), which has a 11 bp extension in MIC III. IR, 96 bp inverted repeat (IR) (GATTAAGCCGGGTTTGTTGTTAAGCCGGGGAACGGTTCGGGGTCTTGGTGGCTGGCCGTGTCCCATGTGGTTTCCCGGCTTAACGTTCCGGGTTAT), that has a 3 bp extension in MIC I and II, a 5 bp extension in MIC III and a 1 bp extension in MIC 1, 2 and 3. IS, insertion sequence. Click here for file

Additional file 3 Comparison of the two genomes of *B. longum *strains DJO10A and NCC2705. (**A**) Mummer3 plot of both genomes. (**B**) ACT4 plot showing the relative locations of mobile elements. Red lines indicate the relative location of elements that are orientated in the same direction. Blue lines indicate elements orientated in opposite directions. Click here for file

Additional file 4 Conserved structure of the *oriC *region. This consists of three clusters, in the two *B. longum *genomes. The DnaA boxes consist of 7 types, designated A to G as follows: Type A (TTATCCACA), Type B (TTGTCCACA), Type C (TTTTCCACA), Type D (TTACCCACA), Type E (TTATCCACC), Type F (TTATTCACA), Type G (TTATGCACA). Click here for file

Additional file 5 Type I and II restriction modification (R-M) systems encoded by the *B. longum *genomes. (**A**) Alignment of the genomic locations encoding a type I R-M system between *B. longum *DJO10 and NCC2705. (**B**) Comparison of a *Sau*3AI-type II R-M system (recognition site, 5'-GATC-3') with analogous R-M systems in other bacteria and (**C**) comparison of a *Eco*RII-type II R-M system (recognition site, 5'-CCWGG-3') with analogous R-M systems in other bacteria. Percentage protein sequence identities compared to *B. longum *DJO10A are indicated in red. Click here for file

Additional file 6 COG categories for all genes in both *B. longum *genomes. Click here for file

Additional file 7 Organization of the 11 different types of oligosaccharide utilization gene clusters (11 in DJO10A and 7 in NCC2705). Red-colored arrows indicate strain DJO10A unique genes. IS, insertion sequence; Hyp, hypothetical protein; Arab, arabinosidase; E, *malE*; F, *malF*; G,*malG*; R, *lacI-*type repressor; K, ATPase of ABC transporter; αGal, α-galactosidase; βXyl, β-xylosidase; Est, esterase; LCFACS, long-chain fatty acid acetyl CoA synthetase; f, fragmented gene; XylT, D-xylose proton symporter; βGal, β-galactosidase; Arab-βGal, arabinogalactan endo-1,4-β-galactosidase; O157, ORF with homolog only in *E. coli *O157; αMan, α-mannosidase; GlycH, glycosyl hydrolase; NAc-Glc, N-acetyl glucosaminidase; UhpB, histidine kinase; RfbA, dTDP-glucose pyrophosphorylase; RfbB, dTDP-D-glucose 4,6-dehydratase; RfbC, dTDP-4-dehydrorhamnose 3,5-epimerase; RgpF, lipopolysaccharide biosynthesis protein; TagG, ABC-type polysaccharide/polyol phosphate export systems, permease component; TagH, ABC-type polysaccharide/polyol phosphate transport system, ATPase component; MdoB, phosphoglycerol transferase; ProP, permease; Acyl-Est, acyl esterase. It should be noted that the glycosyl hydrolase gene in cluster 7 was annotated as isomaltase in the NCC2705 genome annotation. Click here for file

Additional file 8 Nucleotide substitution analysis of all gene homologs between *B. longum *DJO10A and NCC2705, according to the dN:dS ratio. Click here for file

Additional file 9 Substitution ratios of the 52 genes in the positive selection category. Click here for file

Additional file 10 Organization of four predicted LPXTG-type, cell surface anchor proteins in *B. longum *DJO10A. The numbers below the signal peptide boxes indicate the location of signal peptides. The size of the respective proteins is indicated in amino acids. Click here for file

Additional file 11 Loss of the lantibiotic gene cluster from *B. longum *DJO10A-JH1. (**A**) Detection of DJO10A specific gene clusters in *B. longum *DJO10A and its fermentation adapted isolate DJO10A-JH1 by PCR. M, 1 kb DNA ladder (Invitrogen); lane 1, unique region 15; lane 2, unique region 6; lane 3, unique region 9; lane 4, unique region 11; lane 5, unique region 5; lane 6, unique region 7; lane 7, unique region 12; lane 8, 16S rRNA partial gene. The red arrow indicates the lantibiotic encoded unique region 12 that is missing from strain DJO10A-JH1. (**B**) Southern blot analysis using a *lanM *probe and the *Eco*RI-digested genomes of *B. longum *strains DJO10A and DJO10A-JH1. The 1.7 kb *Eco*RI band containing *lanM *is indicated with an arrow. Click here for file

Additional file 12 Growth curves in RCM medium of the four bacteria used in the fecal competitive growth experiments. All bacteria were inoculated at 1% from freshly grown cultures. Black squares, *E. coli *DJOec1; green triangles, *Clostridium difficile *DJOcd1; purple circles, *B. longum *DJO10A-JH1; and blue diamonds, *B. longum *DJO10A. Click here for file

Additional file 13 Primers used in this study. Click here for file
