# Supplementary material for: Comparative genomic analysis of the gut bacterium Bifidobacterium longum reveals loci susceptible to deletion during pure culture growth
Source: BMC Genomics. 2008 May 27;9:247. doi: 10.1186/1471-2164-9-247 (PMC2430713; doi:10.1186/1471-2164-9-247)
Supplement: Additional file 1 — Comparison of serine codon usage between chromosomal and prophage genes in strain DJO10A. [file 1471-2164-9-247-S1.pdf]

		Non-prophage genes		Prophage genes	
Amino acid	Codon	Number	Frequency	Number	Frequency
Serine	UCU	2416	0.35	20	0.18
	**UCC**[a]	**16802**	**2.45**	169	1.53
	UCA	2277	0.33	33	0.30
	UCG	9031	1.32	97	0.88
	AGU	1837	0.27	19	0.17
	**AGC**[b]	8769	1.28	**324**	**2.94**

[a], bold indicates the most frequent codon; [b], AGC is compatible to the extra tRNA_Ser in the prophage genome.
