# Supplementary figures and images for: Comparative genomic analysis of the gut bacterium Bifidobacterium longum reveals loci susceptible to deletion during pure culture growth
Source: BMC Genomics. 2008 May 27;9:247. doi: 10.1186/1471-2164-9-247 (PMC2430713; doi:10.1186/1471-2164-9-247)

**A**

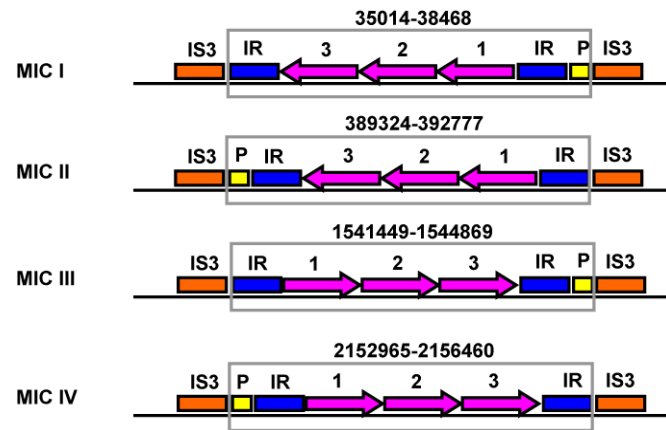

**B**

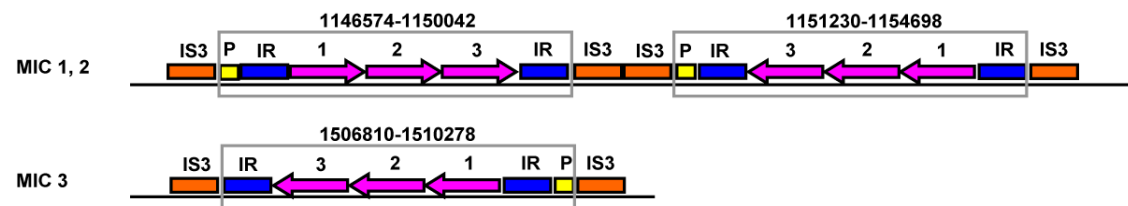

Supplement: Additional file 2 — Organization of mobile integrase cassettes (MIC) in B. longum DJO10A. (A) and NCC2705, (B). Orfs 1, 2 and 3 refer to three contiguous, but different xerC integrase genes. P, a conserved 20 bp palindrome (TTAAACCGACATCGGTTTAA), which has a 11 bp extension in MIC III. IR, 96 bp inverted repeat (IR) (GATTAAGCCGGGTTTGTTGTTAAGCCGGGGAACGGTTCGGGGTCTTGGTGGCTGGCCGTGTCCCATGTGGTTTCCCGGCTTAACGTTCCGGGTTAT), that has a 3 bp extension in MIC I and II, a 5 bp extension in MIC III and a 1 bp extension in MIC 1, 2 and 3. IS, insertion sequence. [file 1471-2164-9-247-S2.pdf]

A

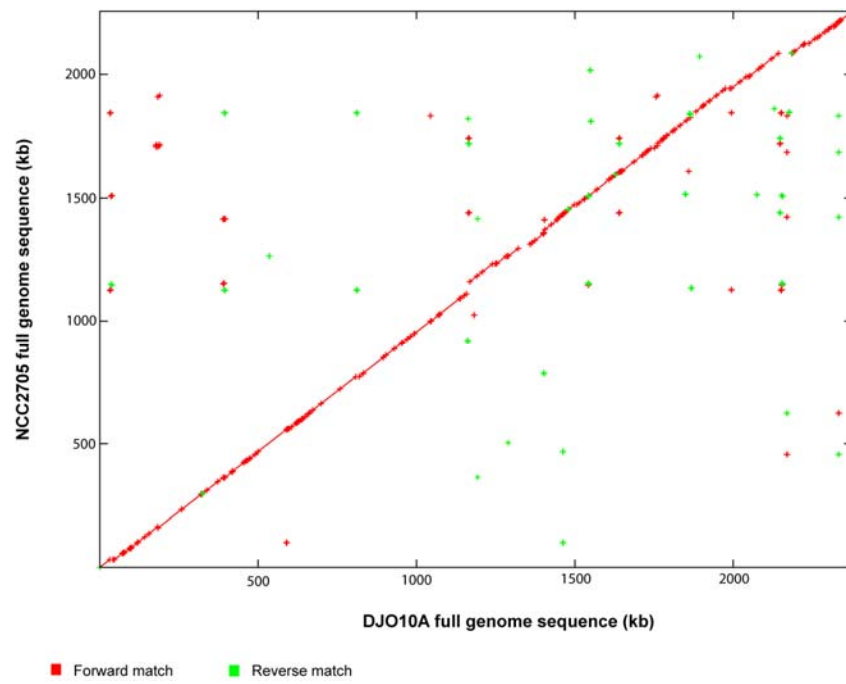

B

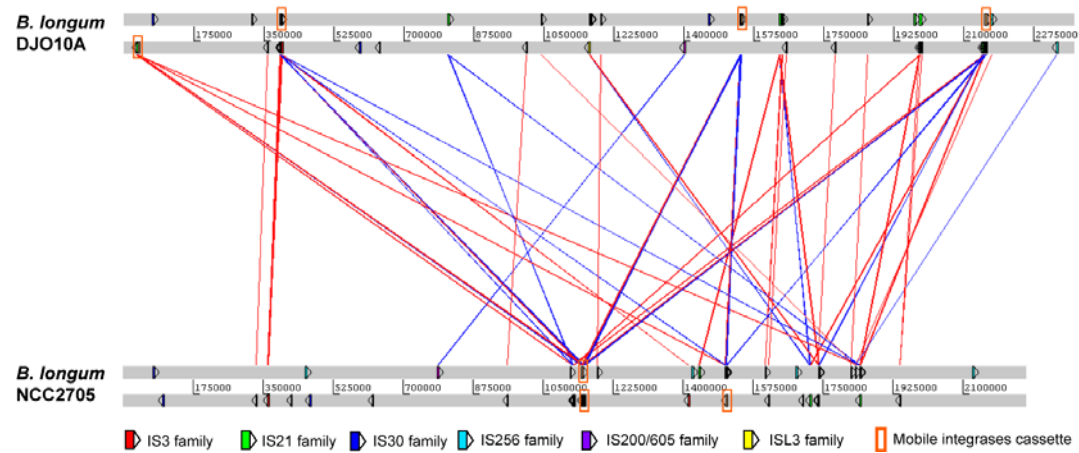

Supplement: Additional file 3 — Comparison of the two genomes of B. longum strains DJO10A and NCC2705. (A) Mummer3 plot of both genomes. (B) ACT4 plot showing the relative locations of mobile elements. Red lines indicate the relative location of elements that are orientated in the same direction. Blue lines indicate elements orientated in opposite directions. [file 1471-2164-9-247-S3.pdf]

A

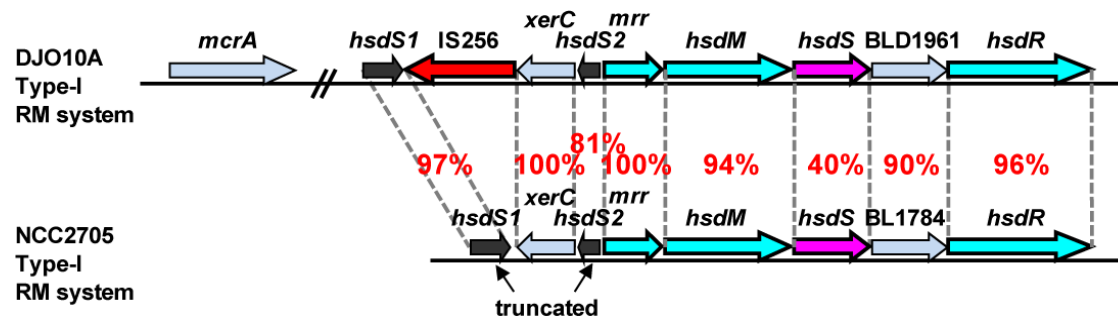

B

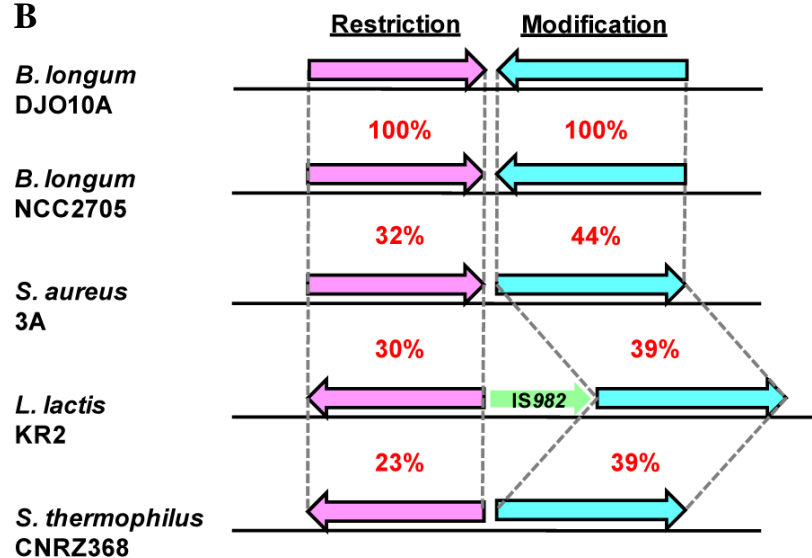

C

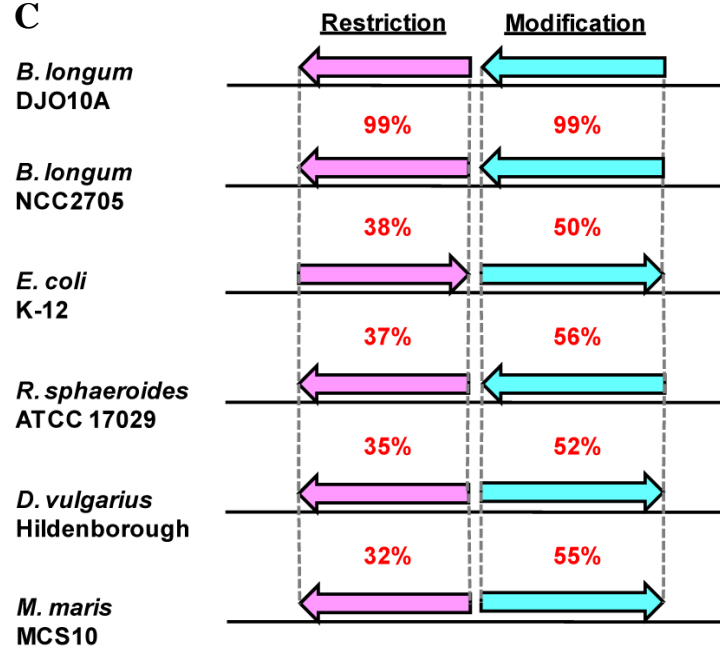

Supplement: Additional file 5 — Type I and II restriction modification (R-M) systems encoded by the B. longum genomes. (A) Alignment of the genomic locations encoding a type I R-M system between B. longum DJO10 and NCC2705. (B) Comparison of a Sau3AI-type II R-M system (recognition site, 5'-GATC-3') with analogous R-M systems in other bacteria and (C) comparison of a EcoRII-type II R-M system (recognition site, 5'-CCWGG-3') with analogous R-M systems in other bacteria. Percentage protein sequence identities compared to B. longum DJO10A are indicated in red. [file 1471-2164-9-247-S5.pdf]

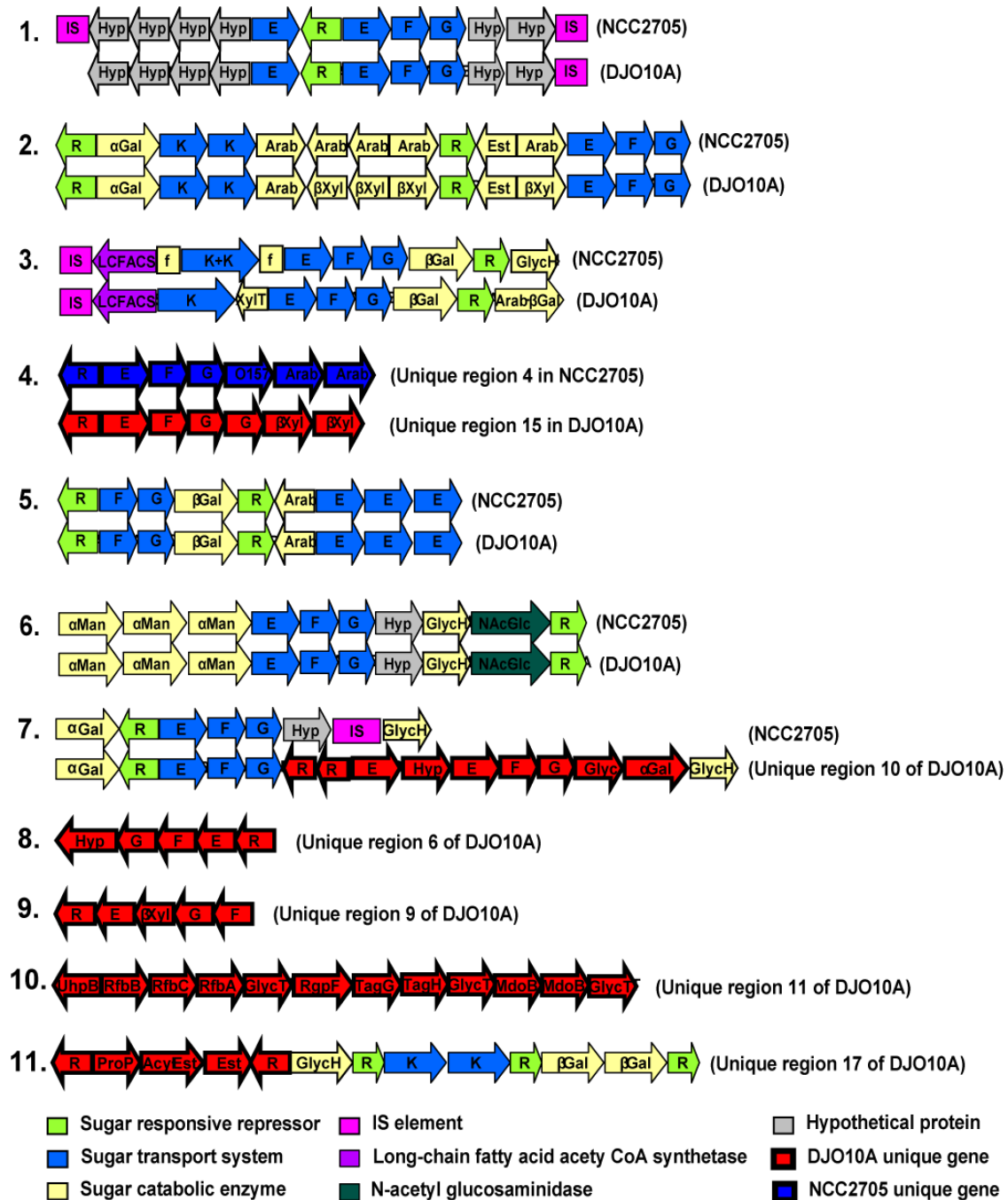

Supplement: Additional file 7 — Organization of the 11 different types of oligosaccharide utilization gene clusters (11 in DJO10A and 7 in NCC2705). Red-colored arrows indicate strain DJO10A unique genes. IS, insertion sequence; Hyp, hypothetical protein; Arab, arabinosidase; E, malE; F, malF; G,malG; R, lacI-type repressor; K, ATPase of ABC transporter; αGal, α-galactosidase; βXyl, β-xylosidase; Est, esterase; LCFACS, long-chain fatty acid acetyl CoA synthetase; f, fragmented gene; XylT, D-xylose proton symporter; βGal, β-galactosidase; Arab-βGal, arabinogalactan endo-1,4-β-galactosidase; O157, ORF with homolog only in E. coli O157; αMan, α-mannosidase; GlycH, glycosyl hydrolase; NAc-Glc, N-acetyl glucosaminidase; UhpB, histidine kinase; RfbA, dTDP-glucose pyrophosphorylase; RfbB, dTDP-D-glucose 4,6-dehydratase; RfbC, dTDP-4-dehydrorhamnose 3,5-epimerase; RgpF, lipopolysaccharide biosynthesis protein; TagG, ABC-type polysaccharide/polyol phosphate export systems, permease component; TagH, ABC-type polysaccharide/polyol phosphate transport system, ATPase component; MdoB, phosphoglycerol transferase; ProP, permease; Acyl-Est, acyl esterase. It should be noted that the glycosyl hydrolase gene in cluster 7 was annotated as isomaltase in the NCC2705 genome annotation. [file 1471-2164-9-247-S7.pdf]

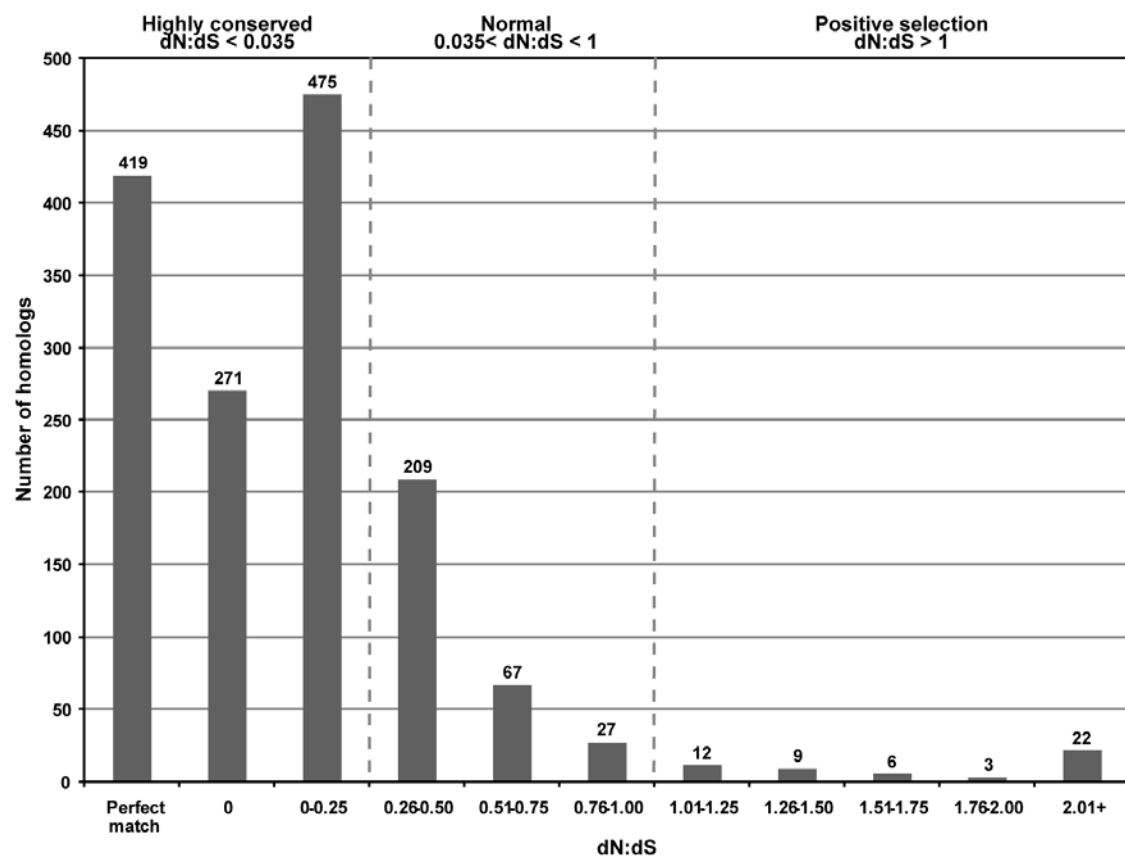

Supplement: Additional file 8 — Nucleotide substitution analysis of all gene homologs between B. longum DJO10A and NCC2705, according to the dN:dS ratio. [file 1471-2164-9-247-S8.pdf]

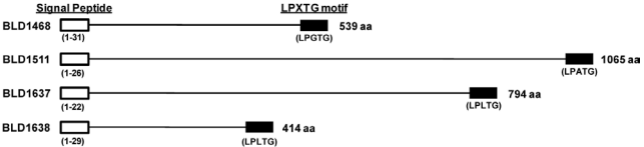

Supplement: Additional file 10 — Organization of four predicted LPXTG-type, cell surface anchor proteins in B. longum DJO10A. The numbers below the signal peptide boxes indicate the location of signal peptides. The size of the respective proteins is indicated in amino acids. [file 1471-2164-9-247-S10.pdf]

**A**

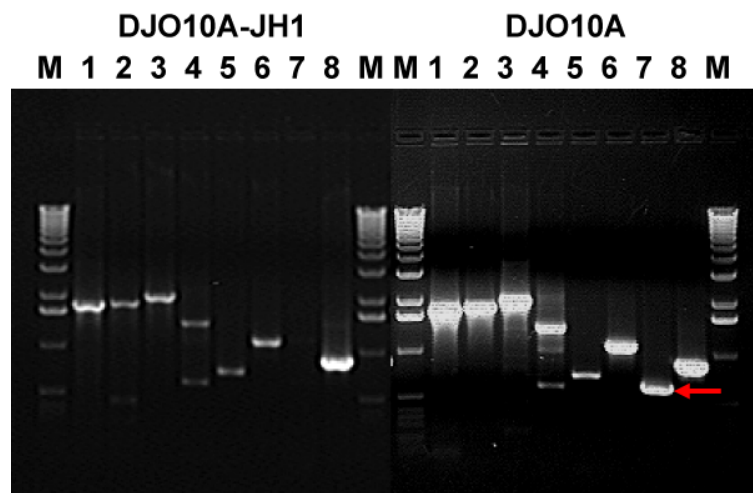

**B**

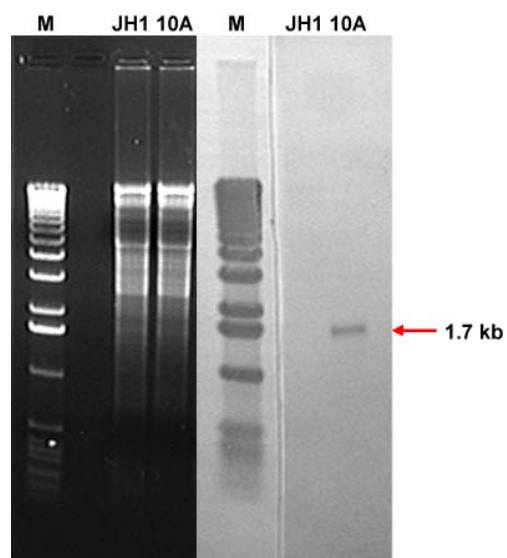

Supplement: Additional file 11 — Loss of the lantibiotic gene cluster from B. longum DJO10A-JH1. (A) Detection of DJO10A specific gene clusters in B. longum DJO10A and its fermentation adapted isolate DJO10A-JH1 by PCR. M, 1 kb DNA ladder (Invitrogen); lane 1, unique region 15; lane 2, unique region 6; lane 3, unique region 9; lane 4, unique region 11; lane 5, unique region 5; lane 6, unique region 7; lane 7, unique region 12; lane 8, 16S rRNA partial gene. The red arrow indicates the lantibiotic encoded unique region 12 that is missing from strain DJO10A-JH1. (B) Southern blot analysis using a lanM probe and the EcoRI-digested genomes of B. longum strains DJO10A and DJO10A-JH1. The 1.7 kb EcoRI band containing lanM is indicated with an arrow. [file 1471-2164-9-247-S11.pdf]

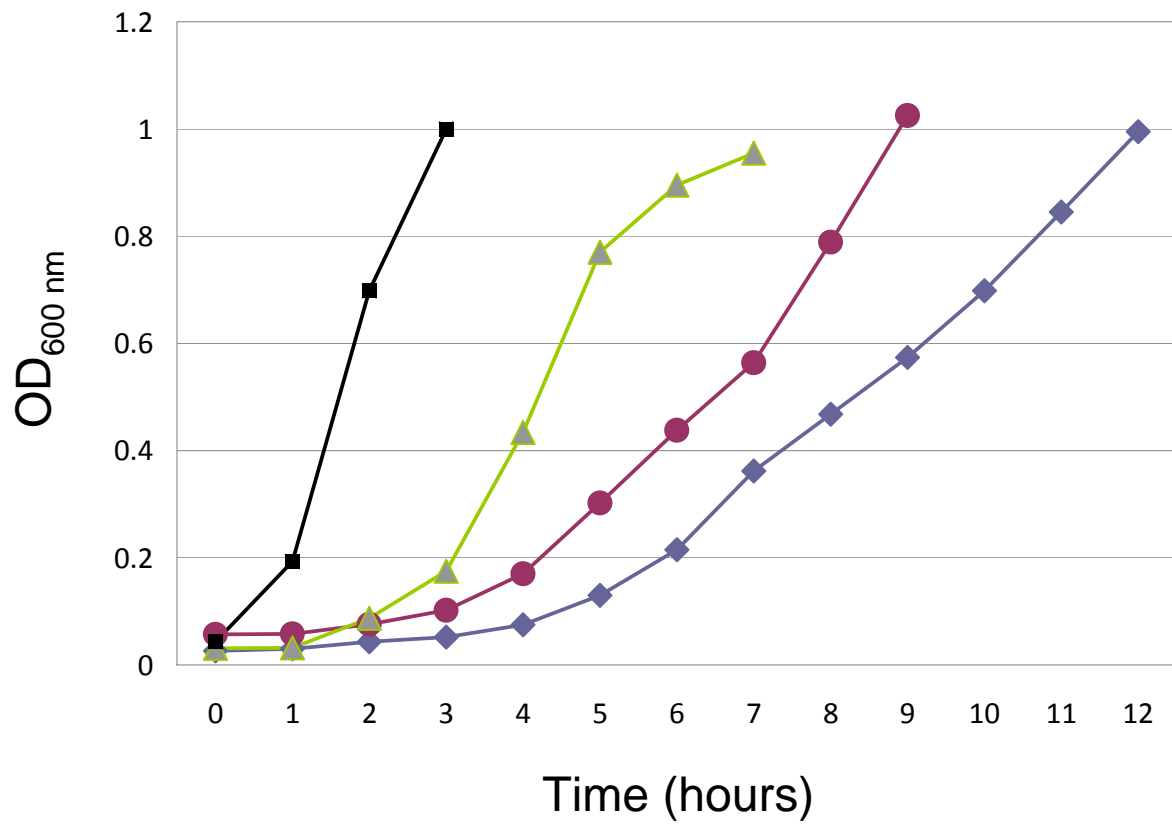

Supplement: Additional file 12 — Growth curves in RCM medium of the four bacteria used in the fecal competitive growth experiments. All bacteria were inoculated at 1% from freshly grown cultures. Black squares, E. coli DJOec1; green triangles, Clostridium difficile DJOcd1; purple circles, B. longum DJO10A-JH1; and blue diamonds, B. longum DJO10A. [file 1471-2164-9-247-S12.pdf]
