# Supplementary material for: Comparative genomic analysis of the gut bacterium Bifidobacterium longum reveals loci susceptible to deletion during pure culture growth
Source: BMC Genomics. 2008 May 27;9:247. doi: 10.1186/1471-2164-9-247 (PMC2430713; doi:10.1186/1471-2164-9-247)
Supplement: Additional file 4 — Conserved structure of the oriC region. This consists of three clusters, in the two B. longum genomes. The DnaA boxes consist of 7 types, designated A to G as follows: Type A (TTATCCACA), Type B (TTGTCCACA), Type C (TTTTCCACA), Type D (TTACCCACA), Type E (TTATCCACC), Type F (TTATTCACA), Type G (TTATGCACA). [file 1471-2164-9-247-S4.pdf]

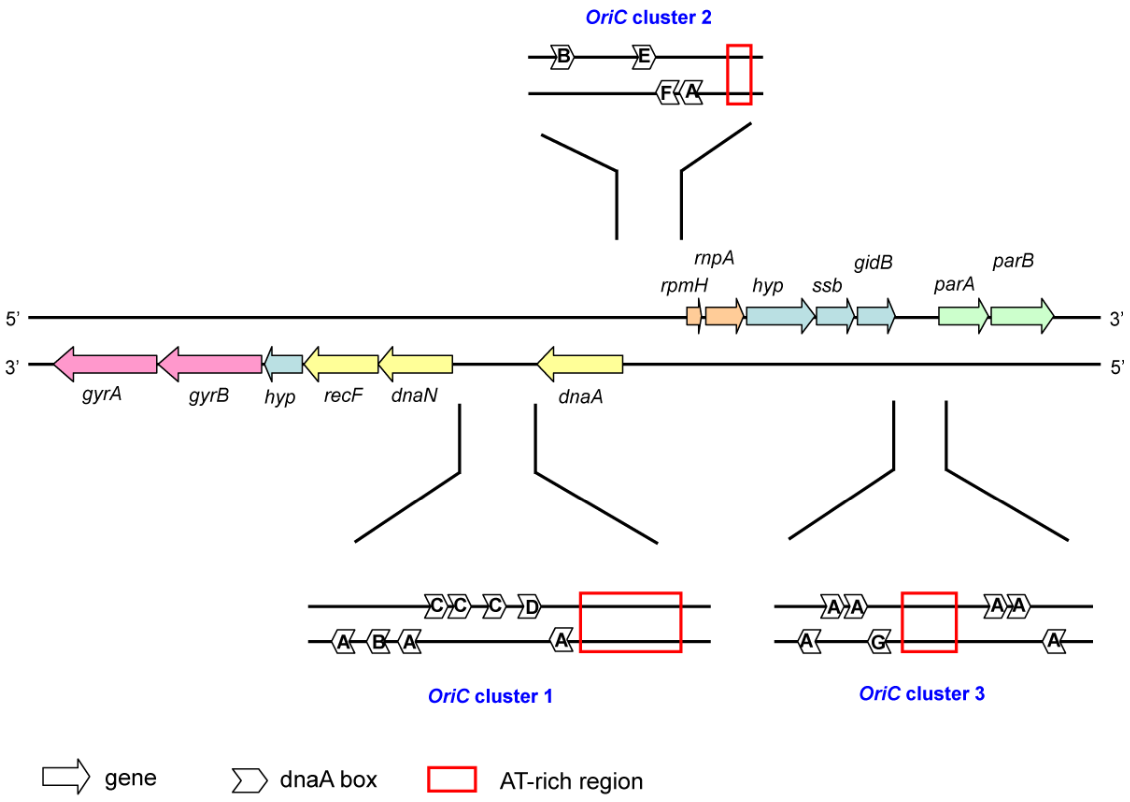

gyrA: DNA gyrase, subunit A
gyrB: DNA gyrase, subunit B
recF: DNA repair protein
dnaN: DNA polymerase III, beta subunit
dnaA: chromosomal replication initiation protein
rpmH: 50S ribosomal protein L34
rnpA: RNase P protein component
ssb: single stranded nucleotide binding protein
gidB: glucose inhibited division protein
parA: chromosomal partitioning protein
parB: cell division protein
hyp: hypothetical protein
