# Supplementary material for: Comparative genomic analysis of the gut bacterium Bifidobacterium longum reveals loci susceptible to deletion during pure culture growth
Source: BMC Genomics. 2008 May 27;9:247. doi: 10.1186/1471-2164-9-247 (PMC2430713; doi:10.1186/1471-2164-9-247)
Supplement: Additional file 6 — COG categories for all genes in both B. longum genomes. [file 1471-2164-9-247-S6.pdf]

Function class	Individual function categories	*B. longum* DJO10A	*B. longum* NCC2705
Information	J: Translation, ribosomal structure and biogenesis	133 (2)[a]	130
	K: Transcription	129 (18)	115 (9)
	L: DNA replication, recombination, and repair	150 (20)	96 (1)
Cellular processes	D: Cell division and chromosome partitioning	22 (2)	23 (1)
	V: Defense mechanisms	48 (5)	48 (3)
	O: Posttranslational modification, protein turnover	51 (2)	50 (2)
	M: Cell envelope biogenesis, outer membrane	68 (8)	67 (10)
	P: Inorganic ion transport and metabolism	56 (2)	54
	U: Intracellular trafficking, secretion	16 (1)	14
	N: Cell motility	3	4
	T: Signal transduction mechanisms	53 (6)	41 (1)
Metabolism	F: Nucleotide transport and metabolism	70 (2)	65 (1)
	G: Carbohydrate transport and metabolism	**188 (32)**	**167 (7)**
	E: Amino acid transport and metabolism	171 (6)	153
	H: Coenzyme metabolism	44	44 (1)
	I: Lipid metabolism	41 (1)	36 (1)
	C: Energy production and conversion	50 (1)	50 (2)
	Q: Secondary metabolites transport and metabolism	6	4
Poorly characterized	R: General function prediction only	167 (20)	161 (15)
	S: Function unknown	524 (142)	405 (63)
Total		1990 (270)	1727 (117)

[a], refers to the number of genes in the unique regions of each genome as defined in the text
