# Supplementary material for: Comparative genomic analysis of the gut bacterium Bifidobacterium longum reveals loci susceptible to deletion during pure culture growth
Source: BMC Genomics. 2008 May 27;9:247. doi: 10.1186/1471-2164-9-247 (PMC2430713; doi:10.1186/1471-2164-9-247)
Supplement: Additional file 9 — Substitution ratios of the 52 genes in the positive selection category. [file 1471-2164-9-247-S9.pdf]

DJO10A	NCC2705	dN:dS	Annotation	Mutation
BLD1991	BL1813	20.0673	Hypothetical protein	Frameshift (NCC2705)
BLD1477	BL1511	14.3257	Hypothetical protein	Frameshift and insertion (NCC2705)
PyrH	*pyrH*	12.9880	Uridylate kinase	Frameshift (NCC2705)
BLD0511	BL0781	12.7529	Hypothetical protein	Frameshift (NCC2705)
BLD0760	BL1007	10.5700	Predicted glycosyltransferase	Frameshift (NCC2705)
ruvB	*ruvB*	9.3214	Holliday junction resolvasome, helicase subunit	
citB	BL1402	7.5024	Response regulator	Deletion (DJO10A)
ppa	*ppa*	6.2668	Inorganic pyrophosphatase	Deletion (NCC2705)
BLD0382	BL1490	6.1532	Hypothetical protein	Frameshift (NCC2705)
BLD1282	BL0491	4.2133	Hypothetical protein	
BLD0801	BL1050	4.0425	Hypothetical protein	
soj	BL1492	3.8930	ATPase involved in chromosome partitioning	
BLD1365	BL0571	3.7661	Predicted esterase	Deletion (NCC2705)
ardA	BL1465	3.6056	Antirestriction protein	
cbiO	BL0049	3.4507	ABC-type cobalt transport system, ATPase component	
BLD0038	BL0026	3.3177	Hypothetical protein	
BLD0376	BL1489	3.2395	Hypothetical protein	Deletion (DJO10A)
dppD	*oppD*	3.1112	ABC-type dipeptide/oligopeptide transport system	
BLD0144	BL0126	3.0474	Hypothetical protein	
BLD1389	BL0595	2.4181	Hypothetical protein	
BLD0109	BL0091	2.3769	Predicted aminoglycoside phosphotransferase	
srtA	BL0676	2.3202	Sortase (surface protein transpeptidase)	Deletion (NCC2705)
BLD0716	BL0962	1.9561	Predicted acyltransferase	
metK	*metK*	1.9085	S-adenosylmethionine synthetase	Frameshift (NCC2705)
BLD1580	BL1246	1.7926	Hypothetical protein	
BLD1774	BL1650	1.7408	Hypothetical protein	
lytE	BL1311	1.7363	LysM repeat	
azlC	BL1669	1.6564	Predicted branched-chain amino acid permease	
glgP	*glgP*	1.5354	Glucan phosphorylase	
BLD1672	BL1553	1.5276	Flagellar basal body P-ring biosynthesis protein	
BLD1399	BL0605	1.5179	Hypothetical protein	
ftsW	*ftsW*	1.4969	Uridylate kinase	
BLD1753	BL1627	1.4775	Predicted transcriptional regulator	
nagA	*nagA*	1.3702	N-acetylglucosamine-6-phosphate deacetylase	
BLD0620	BL0885	1.3620	Predicted acyltransferase	Stop mutation (DJO10A)
DAP2	BL1649	1.3497	Dipeptidyl aminopeptidase/acylaminoacyl-peptidase	
BLD1741	BL1614	1.3341	Hypothetical protein	
topB	*topB*	1.3066	Topoisomerase III	Annotation difference[a] (NCC2705)
topA	*topA*	1.2608	Topoisomerase I	
BLD0571	BL0837	1.2585	Hypothetical protein	
BLD0397	BL1498	1.2350	Hypothetical protein	
wecD	BL1151	1.2166	Histone acetyltransferase HPA2	
pepC	*pepC2*	1.1349	Aminopeptidase C	
sdrC	BL0094	1.1037	Predicted secreted protein containing a PDZ domain	
BLD1612	BL1278	1.0894	ABC-type transport system	
BLD1568	*rmlB1*	1.0865	dTDP-D-glucose 4,6-dehydratase	
BLD0548	BL0810	1.0694	Hypothetical protein	
BLD1455	BL0660	1.0642	Serine/Threonine protein kinase	
BLD0375	BL1488	1.0557	Hypothetical protein	Deletion (NCC2705)
BLD1772	BL1648	1.0402	Hypothetical protein	
BLD1401	BL0607	1.0338	Hypothetical protein	
BLD1983	BL1815	1.0056	Hypothetical protein	

[a], An N-terminal extension in the annotation of TopB in strain NCC2705 does not map to any other TopB sequence.
