# Supplementary material for: Comparative genomic analysis of the gut bacterium Bifidobacterium longum reveals loci susceptible to deletion during pure culture growth
Source: BMC Genomics. 2008 May 27;9:247. doi: 10.1186/1471-2164-9-247 (PMC2430713; doi:10.1186/1471-2164-9-247)
Supplement: Additional file 13 — Primers used in this study. [file 1471-2164-9-247-S13.pdf]

| Target region | Primer | Sequence[b] | Size | Reference |
|---|---|---|---|---|
| **Unique region no.[a]** | | | | |
| Oligo cluster | | | | |
| 15 | OLIGO15-F | 5'-GAAATCCCGAAANACNACC-3' | 1,793 bp | This study |
| | OLIGO15-R | 5'-GTTGCCGATGTTYTGNCC-3' | | |
| 6 | OLIGO6-F | 5'-GTATGTGATGAGCGGNAGY-3' | 1,840 bp | This study |
| | OLIGO6-R | 5'-ACCAACGGATTTYTGNGG-3' | | |
| 9 | OLIGO9-F | 5'-AAGTTCACCGATGARACN-3' | 2,001 bp | This study |
| | OLIGO9-R | 5'-GTAACGCAACGARTAYTCC-3' | | |
| 11 | OLIGO11-F | 5'-TCCCCAACTACATTATHGTNG-3' | 1,419 bp | This study |
| | OLIGO11-R | 5'-TCAACACCATCNGCNACC-3' | | |
| Arsenic cluster | | | | |
| 5 | ARS5-F | 5'-ATTGGCTTATTGCTNACN-3' | 736 bp | This study |
| | ARS5-R | 5'-GACTGCTTCAACTGNAGDATCC-3' | | |
| 7 | ARS7-F | 5'-ACAGTCCCAATACAGTAARACN-3' | 1,125 bp | This study |
| | ARS7-R | 5'-CTCAAAGAAATTAGANGCNCC-3' | | |
| Lantibiotic | | | | |
| 12 | LANT-F | 5'- CGCTATTACACCAGATACG -3' | 646 bp | This study |
| | LANT-R | 5'- GGTAGACATACAGGTTCTCC -3' | | |
| Positive control (16S rRNA gene) | | | | |
| | 16S-F | 5'-CAGCWGCCGCGGTAATWC-3' | 890 bp | (Lane et al., 1985) |
| | 16S-R | 5'-ACGGGCGGTGTGTRC-3' | | |
| **Deletion of lantibiotic operon** | | | | |
| Forward | F3 | 5'- ATCCAACGAGCAAGAACC-3' | | This study |
| Reverse | R3 | 5'-GTGAAATCACCACTACCACC-3' | | |
| **Deletion of MIC III region** | | | | |
| Upstream | MIC-F1 | 5'-CACATCTTGGAACTGCTTGG-3' | | This study |
| | MIC-R1 | 5'-CGTACACCGATGAATGACC-3' | | |
| Downstream | MIC-F2 | 5'-GTTCTTCGTCACCTCCACC-3' | | |
| | MIC-R2 | 5'-AGTAATGTCCCGAATCCTCC-3' | | |
| **IS elements** | | | | |
| IS30 | IS30-F | 5'-GACAAACCCAAGACCCTCC-3' | 352 bp | This study |
| | IS30-R | 5'-CGTGCATATCCCCATTATCC -3' | | |
| IS21 | IS21-F | 5'-GCCCCAAGTACAGTCTATCC-3' | 681 bp | This study |
| | IS21-R | 5'-CAGAACGAACAATCGAACC-3' | | |
| IS256 | IS256-F | 5'-TGTCACAGCAGATTCTACAGG-3' | 719 bp | This study |
| | IS256-R | 5'-CAGCAATTCGTTCACAGC -3' | | |
| ISL3 | ISL3-F | 5'-CGAGATCGTCGAGCTTTCC -3' | 169 bp | This study |
| | ISL3-R | 5'-ATCAGGGCGATGAGGTTGG -3' | | |

[a], as defined in the text ; [b], Y (C/T), R (A/G), H (A/C/T), D (A/G/T), N (A/T/G/C), W (A/T)

## Additional reference

Lane, D.J., Pace, B., Olsen, G.J., Stahl, D.A., Sogin, M.L., and Pace, N.R. 1985. Rapid determination of 16S ribosomal RNA sequences for phylogenetic analyses. *Proc. Natl. Acad. Sci. USA* **82**: 6955-6959.
